# Luminescent property and application values of a new Cd(II) compound in tuberculosis treatment and clinical nursing

**DOI:** 10.1080/15685551.2021.1968113

**Published:** 2021-08-19

**Authors:** Han-Xiao Tao, Jia-Li Jiang

**Affiliations:** Department of Respiratory and Critical Care Medicine, Zhuji Affiliated Hospital of Shaoxing University, Zhuji, China

**Keywords:** Cd(II) compound, hydrothermal synthesis, luminescence, tuberculosis

## Abstract

Through the reaction between HTrz and Cd(NO_3_)_2_ · 4H_2_O, a new Cd(II) compound of [Cd(Trz) _2_] _n_ (**1**, HTrz = 1,2,4-triazole) can be obtained, which has been studied with diffraction analysis by single crystal X-ray as well as powder X-ray diffraction. The structure of **1** can be stable up to 265°C, and the solid samples of **1** emit intense blue luminescence at room temperature. Along with the evaluation of tuberculosis treatment and clinical nursing, related mechanism was also studied here. Firstly, ELISA assay was conducted and the IL-10 and IL-18 released into the alveolar lavage fluid was determined. Apart from this, the real-time RT-PCR was used to reflect surviving gene’s relative expression of *Mycobacterium tuberculosis* after compound treatment.

## Introduction

Because loss caused by the outbreak of infectious diseases is far greater than the cost of prevention, the early prevention of infectious diseases has become a long-term research hotspot [1]. According to the World Health Organization report, in 2015, tuberculosis has taken near2 million lives, and over 10 million people worldwide suffered from tuberculosis in 2016 [2]. How to reduce the number of deaths from tuberculosis by 90% from 2016 to 2035 is a problem facing by all researchers aimed at tuberculosis prevention and treatment. Currently, metal-organic frameworks (MOFs) are attached close attention due to promising topological architectures and functional properties, which brings huge application prospects in many areas, such as storing gas, conducting proton, catalysis, luminescence and magnetism, etc [3–6]. Generally speaking, many reports have demonstrated that the MOFs can be easily synthesized *via* the hydro(solvo)thermal self-assembly reactions of organic ligands bearing multipoint coordination sites and d^10^ transition metal ions [7–9], and the structural diversities and functional properties of MOFs can be realized via tuning or modifying of the organic ligands [10–12]. Obviously, the specific choice of organic ligands with proper sites of flexibility, symmetry and coordination is crucial for us to develop new functional MOFs. As one kind of N-heterocyclic ligand, 1,2,4-triazole has been most commonly used to construct MOFs due to its versatile modes of *μ*_1,2_, *μ*_1,4_, *μ*_1,2,4_ coordination, and most of the 1,2,4-triazole-based MOFs are in combination with anionic bridging coligands [13–17]. In viewing of the good coordination ability and versatile coordination mode of HTrz ligand, in this work, HTrz was combined with Cd(II) ions hydrothermally, as a result in preparing an original Cd(II) compound of [Cd(Trz)_2_]_n_ (**1**, HTrz = 1,2,4-triazole), which features (3,6)-connected flu topology in 3D framework. In addition, the thermal behavior and luminescent property of **1** were also discussed. Furthermore, serial experiments were conducted and the biological activity of the new compound was evaluated.

## Experimental

### Materials and instrumentation

Analytical grade reagents are the starting point for this work, which can be purchased from Jinan Henghua company. An elemental Vario EL III analyzer was prepared to for analysing elements (C, H and N). Additionally, a powder diffractometer of PANalytical X’Pert Pro, which features Cu/Kα radiation (λ = 1.54056 Å) with a 0.05 step, is used to record the Powder X-ray diffraction (PXRD) for analysis. In addition, thermogravimetric analysis was conducted by thermoanalyzer made by a NETSCHZ STA-449C with a temperature rising rate of 10 °C/min in air atmosphere from 30 to 800 °C. The luminescent spectra were measured by a fluorescence spectrophotometer manufactured by Edinburg FLS920 TCSPC under room temperature.

### Synthesis of compound [Cd(Trz)_2_]}_n_ (1)

Firstly, Cd(NO_3_) _2_ · 4H_2_O (0.100 mmol), H_2_O (12.0 mL), HTrz (0.2 mmol), and NaOH (0.5 mmol) was mixed, then this mixture was sealsed into a Parr Teflon-lined stainless steel vessel of 23 ml. This mixture was then kept for 72 h at 180°C. When it cooling down to ambient temperature naturally, transparent blockwise crystals of **1** were separated in 38% yielding based on Cd(NO_3_)_2_ · 4H_2_O. Elemental analysis calcd. for C_4_H_4_CdN_6_ (248.54): C, 19.31; H, 1.61; N, 33.80%. Found: C, 19.28; H, 1.62; N, 33.78%.

### X-ray crystallography

These single crystal data of structure were sampled by room temperature using a computer–recorded Mercury CCD diffractometer, which is equipped with a graphite monochromated Mo–K*α* radiation (*λ* = 0.71073 Å). Through ShelXS structure solution program based on Direct Methods and the ShelXL refinement package based on least-squares minimization, the structure was solved and refined [18]. The crystallographic data of **1** are presented in Table 1.

**Table 1.** The crystal data of compound **1.**

Formula	C_4_H_4_CdN_6_
Fw	248.54
Crystal system	tetragonal
Space group	*I*4_1_/a
*a* (Å)	8.3261(3)
*b* (Å)	8.3261(3)
*c* (Å)	19.4667(12)
α(°)	90
*β*(°)	90
*γ*(°)	90
Volume (Å^3^)	1349.51(13)
*Z*	8
Density (calculated)	2.447
Abs. coeff. (mm^−1^)	3.169
Total reflections	4796
Unique reflections	752
Goodness of fit on *F^2^*	0.929
Final *R* indices [*I*> 2sigma(*I*^2^)]	*R*= 0.0156, *wR*_2_ = 0.0556
*R* (all data)	*R*= 0.0160, *wR*_2_ = 0.0562
CCDC	2088168

### ELISA assay

In this research, the ELISA detection kit is used to test the substance of the IL-10 and IL-18 released into the alveolar lavage fluid after compound treatment. Only limited change was involved in conduction under the guidance of instructions. Briefly speaking, 50 BALB/c mice used in the present study were obtained from the Changchun Jingcheng Model Animal Technology Co., Ltd. All the animal were kept with 20–25°C, which is the standard reference, providing normal food and water. Then, Mycobacterium tuberculosis (10^8^ CFU) was planted into the mice to set the tuberculosis animal model. This compound was then given at 1, 2 and 5 mg/kg as treatment. Finally, the alveolar lavage fluid of the animal was recorded and ELISA kit was used to test the inflammatory cytokines released into the plasma.

### Real time RT-PCR

After compound treatment, the surviving gene’s relative expression of Mycobacterium tuberculosis was recorded by real-time RT-PCR. This preformation was performed strictly in accordance with the manufactures’ instruction with some modifications. Shortly, the Mycobacterium tuberculosis (10^8^ CFU) was injected into the mice to build tuberculosis animal model. The compound was then given at the concentration of 10, 20 and 50 μg/mL as treatment. Next, the total RNA in the bacterial cells was extracted with TRIZOL reagent. Due to the reverse transcripting into cDNA, the concentration of the total RNA was recorded. In the end, the surviving gene’s relative expression of Mycobacterium tuberculosis was determined, with *gapdh* used as control sample.

## Results and discussion

### Crystal structure of compound 1

**Figure 1.**
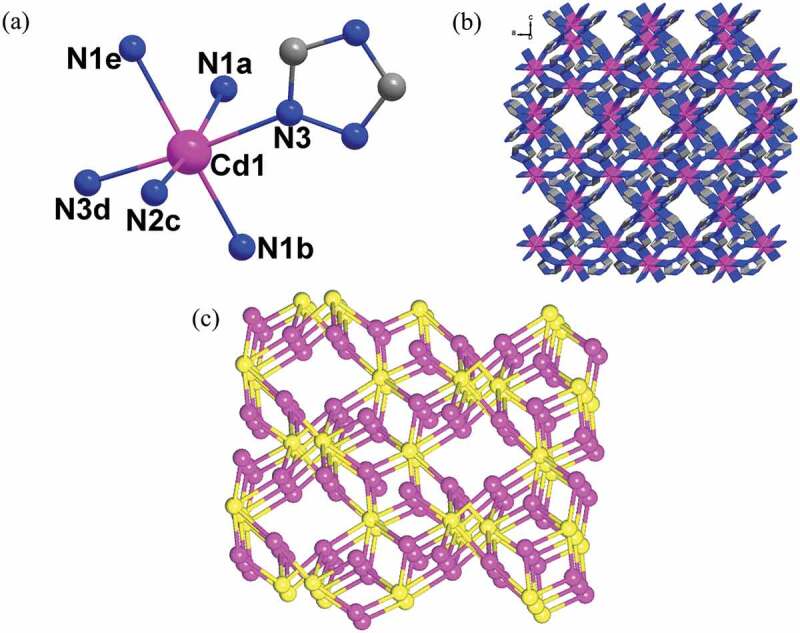
(a) The coordination environments of Cd(II) ion. (b) The 3D framework of **1**. (c) Schematic representation of (3,6)-connected flu topological network for **1.**

Single crystal structural analysis showed that the structure of **1** crystallizes in the tetragonal space group of *I*4_1_/a, and displays a 3D framework representing a (3,6)-connected **flu** topological network. The asymmetric unit of **1** is constituted by one Cd(II) ion and one *μ*_3_-Trz ligand. As shown in Figure 1(a), each Cd(II) center shows s slightly distorted octahedral geometry that is defined by six nitrogen atoms from six independent *μ*_3_-Trz ligands. The Cd-N bond lengths and N-Cd-N bond angles are in the range of 2.3499(16)-2.4082(17) Å, 80.85(9)-168.33(6)º, respectively. In **1**, each Trz ligand coordinates with three Cd(II) ions in a *μ*_3_ bridging mode using its three N donor sites. In such a connecting manner, the *μ*_3_-Trz ligands connect the adjacent Cd(II) ion to afford a 3D framework with rhombus windows viewed along the b axis (Figure 1(b)). By analysis of this 3D framework, each Trz ligand connects with three Cd ions and each Cd(II) ion is six-coordinated with six adjacent Trz ligands, so they can be topologically reduced into 3-, 6-connected nodes, respectively. Thus, the 3D framework of **1** can be seen as a binodal (3,6)-connected network with **flu** topology and the short Schläfli symbol of this net is {4.6^2^}_2_{4^2^.6^7^.8^6^} via the calculation of Topospro software (Figure 1(c)). According to the reports, it can be found that most of the Cd-Trz-based compounds are constructed with the help of auxiliary anions [11–15]. As far as is known, Trz ligand and Cd(II) ions constructed such Cd(II) compound as the first example.

### Powder X-ray diffraction pattern (PXRD) and thermogravimetric analysis (TGA)

**Figure 2.**
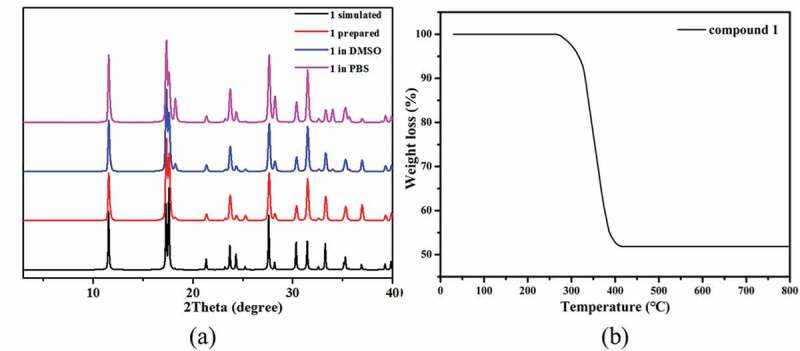
(a) The PXRD patterns for **1**. (b) The TGA curve of **1.**

The phase purity of **1** was demonstrated *via* the characterization of PXRD experiment. The pattern measured for the bulk samples agreed well with simulated group generated by data from single crystal diffraction (Figure 2(a)), showing that the samples of **1** are in phase of pure. Considering the following bioactivity tests, it is necessary to study the framework stability of complex **1** in the dispersing solvent DMSO and the simulated *in vivo* condition phosphate buffered saline (PBS). Due to complex **1** could not be dissolved in the organic solvents and water, we used its stock solution in the following bioactivity tests according to the literature methods [19,20]. With this in mind, about 100 mg of the as-prepared crystalline samples of **1** were was taken in a mortar. It was then being ground manually for 30 min by using a pestle. The produced fine powders were soaked in 20 mL PBS or DMSO and subjected to the ultrasonic treatments for 2 hours to obtain the well-dispersed solution. After standing for 2 days, the fine powders could be recovered via centrifugation and their PXRD measurements show that the PXRD profiles of the treated samples show good matches with those of the as-prepared samples, reflecting their good stability in the above conditions.

Additionally, the TGA experiment for **1** was carried out to investigate the thermal stability under air atmosphere between 30°C and 800°C (Figure 2(b)). By the TGA curve of **1**, we can observe that there is a stable platform from 30°C to 265°C, indicating that the framework of **1** can be stable up to 265°C. After 265°C, the framework began to decompose with a significant weight loss continued to 416°C. The remaining weight of 51.78% corresponds to the formation of CdO (51.67%).

### Photoluminescent property of 1

**Figure 3.**
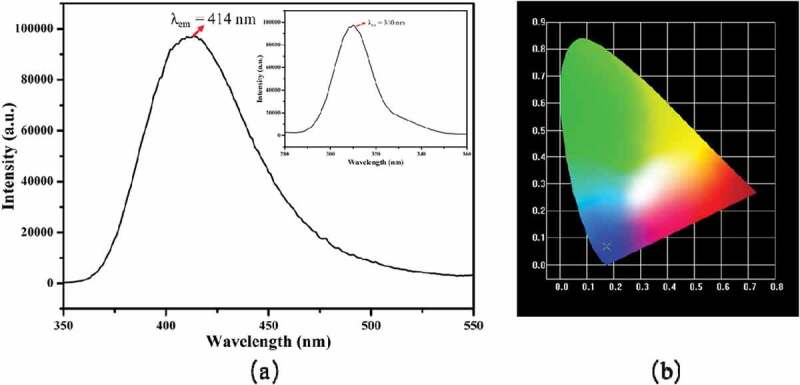
(a) The luminescent emission spectrum of **1** by temperature of nature room (Inset is the exciting spectrum of **1**). (b) The CIE chromaticity diagram for **1.**

We measured luminescent spectrum of **1** by room temperature. As shown in Figure 3(a), an intense emission band for **1** is observed at 414 nm upon 310 nm of exciting. In addition, the luminescent properties of the free HTrZ ligand were examined under the same conditions, and found that the free HTrz ligand is non-fluorescent in the range of 300–800 nm upon excitation of 280–250 nm. Theoretically speaking, the highest occupied molecular orbitals (HOMOs) of **1** are probably contributed by the π-bonding orbitals of Trz ligands, whereas the lowest occupied molecular orbitals (LUMOs) of **1** are mostly contributed by the Cd-N σ*-antibonding orbitals accounted more by those of metal ions. Thus, the emission band of **1** can be counted as ligand-to-metal charge transfer (LMCT) [21,22]. From the CIE chromaticity diagram, it can be found that the CIE chromaticity coordinate of **1** is at (0.1726, 0.0686), which locates in the blue light region, indicating that **1** may be served as an excellent blue luminescent material.

### Compound significantly reduce the releasing of the IL-10 and IL-18 into the alveolar lavage fluid

**Figure 4.**
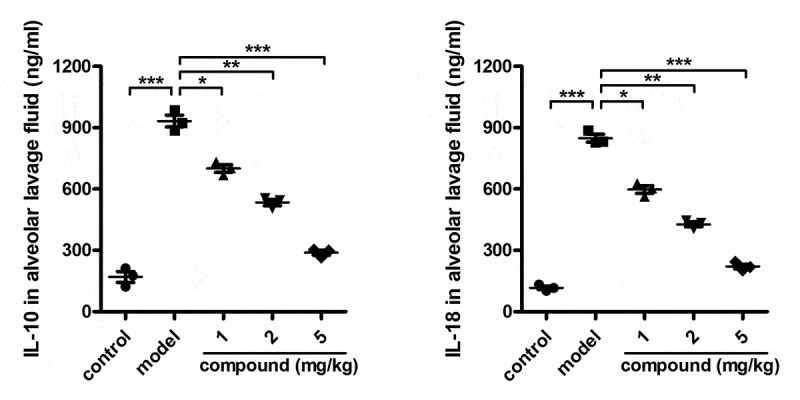
The significantly reduced level of the IL-10 and IL-18 released into the alveolar lavage fluid by the compound. The Mycobacterium tuberculosis was injected to the animal, to develop a tuberculosis animal model, and a concentration of 1, 2 and 5 mg/kg of this compound was used for treatment. ELISA assay was used to determine the level of the IL-10 and IL-18 released into the alveolar lavage fluid

The next step of synthesis of this original compound is its treatment activity. Thus, the ELISA assay was firstly conducted to determine the levels of IL-10 and IL-18 released into the alveolar lavage fluid. The results in Figure 4 presented a much higher level of IL-10 and IL-18 in the model group, compared with the control group. However, after this new compound is treated, the level of IL-10 and IL-18 released into the alveolar lavage fluid was reduced dose dependently.

### Compound obviously inhibited the relative expression levels of survival gene of Mycobacterium tuberculosis

**Figure 5.**
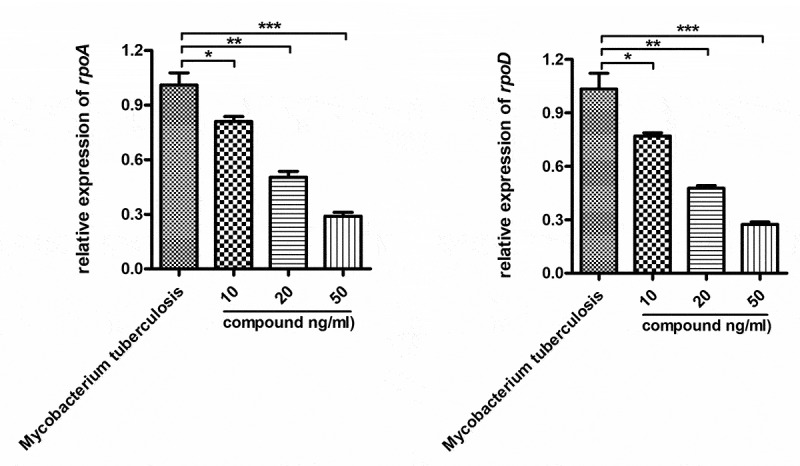
The obviously repressed relative expression levels of survival gene of Mycobacterium tuberculosis by the compound. The Mycobacterium tuberculosis were harvested and treated by the compound with different concentrations. The relative expression levels of survival gene of Mycobacterium tuberculosis were measured with RT-PCR in real time

This study has proven that this compound has good repressing effect on reducing of IL-10 and IL-18 content in the alveolar lavage fluid. Nonetheless, if this compound has an influence on the relative expression levels of surviving gene of *Mycobacterium tuberculosis* was still need to be explored. However, the compound’s effect on the relative expression levels of survival gene of Mycobacterium tuberculosis is yet to be measured. So, a real-time RT-PCR was going to be carried out in next step of research. Figure 5 tells us that, the relative expression levels of survival gene of Mycobacterium tuberculosis could be obviously reduced by the new compound. This inhibition exhibited a dose relationship.

## Conclusion

By this study, we reported an original Cd(II) compound, which is based on trinuclear [Cd_3_(COO)_5_] clusters that represents a 3-fold interpenetrated **bsn**-type topological network. For the synthesis of **1**, tetramethylammonium cations play important roles not only acting as templates but also acting as balance charges. Interestingly, compound **1** also shows good photocatalytic activity despite of the MV degrading due to the irradiation of UV light. The results of the ELISA suggested this compound can repress IL-10 and IL-18 flowing into the alveolar lavage fluid. In addition to this, the surviving gene’s expressing levels of Mycobacterium tuberculosis after compound treatment were also reduced dose-dependently. In conclusion, this compound is proven to have great potential competence in tuberculosis treatment by reducing the expression levels of survival gene of Mycobacterium tuberculosis.

## Data Availability

The data used to support the findings of this study are included within the article.
